# Synergistic regulation of selenium yeast and vitamin E on the rumen microbiota-VFA-liver axis in lambs

**DOI:** 10.1186/s12866-026-04785-3

**Published:** 2026-03-19

**Authors:** Shangwu Ma, Yuzhu Sha, Xiaoyong Ma, Haibo Wang, Shengguo Zhao, Ting Jiao

**Affiliations:** 1 College of Animal Science and Technology, Gansu Agricultural University, Lanzhou, 730070 China; 2 College of Pratacultural Science, Gansu Agricultural University, Lanzhou, 730070 China

**Keywords:** Immunity, Antioxidant, Microbiota, VFA, Transcriptome

## Abstract

**Background:**

In intensive livestock production, various environmental and management stressors often compromise the oxidative and immune homeostasis of lambs, thereby impairing their growth performance and health. Selenium and vitamin E are well-established antioxidant nutrients; but their combined regulatory effects via the rumen-liver axis remain insufficiently understood. To investigate this, twenty-four healthy lambs (average body weight: 20.39 ± 1.52 kg) were randomly assigned to four dietary treatment groups (*n* = 6 per group) and housed individually. The groups were as follows: a control (CON) group fed a basal diet; an SY group fed the basal diet supplemented with 0.6 mg/kg selenium yeast (SY); a VE group fed the basal diet supplemented with 200 IU/kg vitamin E (VE); and a MIX group fed the basal diet supplemented with both 0.6 mg/kg SY and 200 IU/kg VE. This study aimed to elucidate the underlying regulatory pathways of the rumen-liver axis by assessing lamb growth performance, immune function, antioxidant capacity, rumen fermentation parameters, and liver transcriptome profiles, and by analyzing the interrelationships among these variables.

**Results:**

Lambs in the MIX group exhibited significantly higher average daily gain, serum concentrations of growth hormone and immunoglobulins, and enhanced serum antioxidant capacity compared to other groups (*P* < 0.05). Concurrently, the rumen environment was notably improved, characterized by reduced pH and ammonia–nitrogen concentration, increased volatile fatty acid (VFA) production, and an elevated abundance of beneficial bacteria such as Firmicutes and Prevotella. Liver transcriptomic analysis further revealed that differentially expressed genes (DEGs) were significantly enriched in pathways related to immune and antioxidant functions, notably arachidonic acid metabolism. Furthermore, correlation analysis suggested that rumen microbiota influence hepatic gene expression profiles through modulation of VFA metabolism.

**Conclusion:**

The findings of this study demonstrate that the combined dietary supplementation of SY and VE enhances antioxidant and immune functions, and synergistically improves the growth performance and health status of lambs. These beneficial effects are closely associated with alterations in a "rumen microbiota–VFA–liver" axis.

## Introduction

The healthy and rapid growth of fattening lambs is a primary objective of modern, efficient sheep production. However, under intensive farming systems, stressors such as early weaning, high-concentrate diets, and environmental challenges frequently disrupt the oxidative and immune homeostasis of lambs. This imbalance compromises growth performance and constrains both production profitability and animal welfare [1, 2]. Therefore, developing safe and effective nutritional strategies to enhance the stress resilience and support the healthy development of lambs has become a significant focus in animal nutrition research.

Selenium yeast (SY), an organic selenium source with high bioavailability and low toxicity, plays a crucial role in antioxidant enzymes such as glutathione peroxidase (GSH-PX) and contributes to redox regulation [3]. Recent studies indicate that SY can significantly alleviate oxidative stress in ruminants. Maurya [4] reported that SY markedly reduced oxidative and metabolic stress in heat-stressed Barbari goats, lowering lipid peroxidation and enhancing overall antioxidant status. Furthermore, SY has been shown to optimize rumen fermentation and modulate microbial composition [5], thereby indirectly improving nutrient digestion and utilization. Vitamin E (VE), a fat-soluble antioxidant, effectively scavenges free radicals, protects cell membrane integrity, and exhibits anti-inflammatory and immunomodulatory properties [6]. Previous research has demonstrated that the combined supplementation of SY and VE can enhance growth performance, improve antioxidant and immune functions, and promote rumen fermentation in ruminants [7, 8]. However, the comprehensive regulatory mechanisms underlying their synergistic effects in lambs, particularly those mediated via the "rumen-liver" axis, remain to be fully elucidated. The rumen is the primary site for nutrient metabolism in ruminants; its microbial ecosystem, fermentation efficiency, and morphological development directly determine digestive and absorptive capacity [9, 10]. The liver, as the central organ for metabolism and detoxification, is also a key target for the physiological functions of both selenium and VE [11, 12]. We hypothesize that SY and VE may synergistically enhance lamb health and growth performance by modulating rumen fermentation patterns and microbial composition to promote the efficient production and utilization of volatile fatty acids (VFAs), while concurrently upregulating hepatic pathways involved in antioxidant defense and immune modulation.

Based on this rationale, this study utilized newly weaned male F1 crossbred Hu lambs as the experimental model. The lambs were administered diets supplemented with SY and VE, both individually and in combination, to systematically evaluate the effects of these additives on growth performance, serum immunological and antioxidant parameters, rumen fermentation characteristics and tissue morphology, microbial community structure, and the liver transcriptome. The objective was to elucidate the synergistic mechanisms through which selenium yeast and vitamin E enhance stress resilience and promote healthy growth in lambs, thereby providing a theoretical foundation and technical framework for precision nutrition and sustainable, health-oriented ruminant production.

Based on this, this study took Hu sheep generation weaned male lambs as the subjects. By adding SY and VE alone and in combination to the diet, the effects of these additives on the growth performance, blood immunity and antioxidant indicators, rumen fermentation characteristics and tissue morphology, microbial community structure, and liver transcriptome of the lambs were systematically evaluated. The aim is to reveal the synergistic mechanism of yeast selenium and vitamin E in enhancing the stress resistance and promoting healthy growth of lambs, providing theoretical basis and technical support for precise nutrition and green and healthy breeding of ruminants.

## Material and method

### Experimental design

Twenty-four healthy and well-in condition weaned male lambs were selected for the experiment, with an average body weight of (20.39 ± 1.52) kg. All the lambs are sourced from a large-scale and well-managed farm in Linxia City, Gansu Province. Before the experiment began, the lambs had completed routine deworming, brucellosis testing and vaccination to ensure a consistent initial health status. Twenty-four lambs were randomly divided into 4 groups according to their weight, with 6 lambs in each group, and were raised in separate pens. CON group: Basal diet, SY group: basal diet + 0.6 mg/kg yeast selenium, VE group: basal diet + 200 IU/kg vitamin E, MIX group: basal diet + 0.6 mg/kg yeast selenium + 200 IU/kg vitamin E. The addition amount was in accordance with the studies of Suganthi [13] and Nurlatifah [14]. The experiment consists of a 7-day pre-test period and a 60-day formal test period. The automatic feeding trough is used to ensure free feeding and drinking. During the experiment, the environmental conditions, feeding procedures and daily management of each group remained consistent. The basic diet used in the experiment was formulated in accordance with the "Lamb Rearing Standards of the Ministry of Agriculture of the People's Republic of China". The specific formula and nutritional levels are shown in Table 1.

**Table 1:** Basic diet formulation

Ingredient	content(%)	Nutrient level (%)	
Corn stalk	15.00	digestive energy DE/(MJ/kg)	10.21
Corn	32.00	CP	14.81
Wheat bran	6.00	EE	3.50
Soybean meal(43%)	6.00	Ash	9.00
Sunflower seed shells	12.00	CF	14.60
Spraying corn skin	8.00	NDF	33.20
DDGS	5.00	ADF	21.50
NaHCO3	0.60	Ca	1.30
Molasses	5.00	TP	0.60
Pumpkin Seed Meal	5.20	NaCl	0.50
Limestone	0.60		
Soybean oil	0.60		
Premix^a^	4.00		
Total	100.00		

^a^Composition (per kg of dry matter): 100,000–500 000 IU of vitamin A, 50,000–200 000 IU of vitamin D3, Fe 1500–7000 mg, Cu 300–750 mg, Mn 1000–5000 mg, Zn 1500–4000 mg, I 20–30 mg, Co 8–35 mg

In this study, to ensure uniform distribution and stable addition, selenium yeast and vitamin E were uniformly mixed into feed ingredients and subsequently processed into whole mixed granular feed through granulation. The high temperature during granulation inactivates live yeast without special protection; therefore, the observed effects in this study should be attributed to the stable organic selenium form in yeast selenium, vitamin E, and other potential yeast metabolites, rather than the action of live yeast cells [15]. Based on this mechanism and their good water solubility/dispersibility, these two additives can be flexibly supplemented via complete granular feed or drinking water in subsequent farming practices to accommodate different feeding management models.

### Sample collection

After the trial period ended, the lambs fasted for 12 h. Blood samples were collected from the jugular vein, centrifuged at 3500 r/min and 4 °C for 10 min, and the serum was separated. After it was stored at −80 °C for the determination of hormones, antioxidants and immune indicators. Blood samples were collected, after which the lambs were slaughtered for tissue collection. Slaughter was conducted humanely at a licensed abattoir in full compliance with the national standard NY/T 3469–2019, which mandates effective stunning to ensure unconsciousness prior to exsanguination. samples were aseptically collected by the research team members. Liver sample: Aseptically collect left lobe tissue of the liver (0.5–1 g), immediately place it in a cryotube containing RNA protective solution and store it at −80 °C for transcriptome sequencing; Rumen tissue: Rumen abdominal capsule tissue (1 cm^3^) was collected and fixed with 4% paraformaldehyde for 24 h for tissue morphology analysis; Rumen fluid: Collect the contents of the rumen abdominal capsule, filter them through 4 layers of sterile gauze, and then aliquot: ① Measure the pH on-site with a portable pH meter (Leici PHS-3C) (repeat three times and take the average value); ② Store at −2℃ for the determination of volatile fatty acids (VFAs) and NH₃-N; ③ Store at −80℃ for microbial DNA extraction.

### Determination of growth performance

At the beginning and end of the formal trial period, sheep in each group were fasting for 12 h and then weighed (with an accuracy of 0.01 kg). The feed consumption at each stage was recorded, and the average daily gain, average daily feed intake and feed-to-weight ratio were calculated.

- Average daily gain (ADG) = (final weight - initial weight)/number of feeding days
- Average daily feed intake (ADFI) = stage feed intake/number of feeding days
- Material-to-weight ratio (F/G) = ADFI/ADG.

### Serum hormone determination

The levels of growth hormone (GH), glucagon (GCG) and insulin growth factor-1 (IGF-1) were determined by ELISA. The operation strictly followed the instructions of the kit. The absorbance at 450 nm was measured by the microplate reader (Thermo Multiskan FC), and the concentration was calculated by the standard curve method (n = 6).

### Antioxidant and immunoglobulin indicators

The total antioxidant capacity of serum (T-AOC), glutathione peroxidase (GSH-PX), superoxide dismutase (SOD), malondialdehyde (MDA), and catalase (CAT) indicators were determined by colorimetry. The levels of IgA, IgG and IgM in serum were determined by ELISA, with quantitative analysis at 450 nm absorbance (n = 6).

### Characteristics of gastric juice fermentation

NH₃-N, Take 5 mL of rumen fluid, add 10 mL of 0.2 mol/L HCl and mix well. Centrifuge at 3500 r/min for 10 min, and measure the supernatant by colorimetry at 420 nm [16]. VFA, Take 1 mL of rumen fluid, add 0.2 mL of 25% metaphosphoric acid (containing 0.8% tobacic acid), centrifuge at 12,000 r/min for 10 min, and the supernatant is filtered through a 0.22 μm filter membrane. Then, determine it by gas chromatography (Shimadzu GC-2010 Plus). Chromatographic conditions DB-FFAP capillary column (30 m × 0.25 mm × 0.25 μm), column temperature 40℃ (3 min) —5℃/min to 180℃ (5 min), detector (FID) 250℃, injection port 220℃, carrier gas (N₂) flow rate 1.0 mL/min The injection volume is 1 μL (split ratio 10:1) [17].

### Preparation and observation of HE sections of rumen tissue

The fixed rumen epithelial tissue was dewatered, paraffin embedded, sectured (5 μm), stained with HE, observed under an optical microscope (Nikon Eclipse Ni-U), and the papillary length, width and muscular layer thickness were determined using Image Pro Plus 6.0 software (10 fields were randomly selected for each sample). Repeat three times and take the average.

### Rumen microbial 16S rRNA sequencing

DNA extraction The total microbial DNA of the tumor fluid was extracted using the MN NucleoSpin 96 Soil kit. The DNA concentration (≥ 50 ng/μL) and purity (A260/A280 = 1.8–2.0) were detected by NanoDrop 2000, and the integrity was verified by 1% agarose gel electrophoresis. PCR amplification PCR amplification was performed on the V3-V4 region of the 16S rRNA gene with primers 338 F (5 '-ACTCCTACGGGAGGCAGCAG-3') and 806R (5 '-GGACTACHVGGGTWTCTAAT-3'). The reaction system (25 μL): 2 × Taq Plus Master Mix 12.5 μL, upstream and downstream primers (10 μmol/L) each 0.5 μL, DNA template 1 μL, enzyme-free water 10.5 μL. Reaction procedure: Pre-denaturation at 95℃ for 5 min, 30 cycles (95℃ for 30 s, 55℃ for 30 s, 72℃ for 30 s), final extension at 72℃ for 10 min. Sequencing and analysis, After the PCR products were purified, they were sequenced on the Illumina MiSeq platform (2 × 300 bp double-ended sequencing). After merging the original data with FLASH v1.2.7, filtering with Trimmomatic v0.33, and de-chimerization with UCHIME v4.2, Usearch v11.0 clustered OTUs with 97% similarity and annotated species based on the Silva 138 database. Bioinformatics analysis on BMKCloud (www.biocloud.net) was used to analyze Alpha diversity (Chao1, Shannon, Simpson, ACE index) and Beta diversity (PCoA analysis),the confidence interval is 95%, the Metastats software was used to test the differences in species abundance between groups [18]. 

### Liver transcriptomic analysis

RNA extraction: Total RNA from liver tissue was extracted using TRIzol reagent. The quality was verified by 1% agarose gel electrophoresis (28S/18S≈2:1) and NanoDrop 2000 (A260/A280 = 1.8–2.0, A260/A230 > 1.5). Library construction and sequencing For each sample, 1 μg of RNA was taken. The cDNA Library was constructed using the Illumina TruSeq Stranded mRNA Library Prep Kit and sequenced using the Illumina NovaSeq 6000 platform (2 × 150 bp double-ended sequencing). Data analysis: The Fastp software filtered the raw data to obtain Clean reads. HISAT2 was aligned to the sheep reference genome (Oar_rambouillet_v1.0), and StringTie was used to calculate the gene expression level (FPKM value). Bioinformatics analysis on BMKCloud was used to analyze DESeq2 screens differentially expressed genes (DEGs), with the criteria being log₂FC ≥ 2 and *P* < 0.05. Functional enrichment analysis was conducted between the GO database and the KEGG database, and Fisher's exact test determined significance (*P* < 0.05) [19].

### Statistical analysis

Statistical analysis was conducted using IBM SPSS Statistics 26.0 software: Growth performance, hormones, antioxidants, immunity and rumen fermentation parameters were analyzed by one-way ANOVA. Duncan’s multiple comparison tests were used to test the differences between groups (*P* < 0.05) was considered significant, it was considered extremely significant (*P* < 0.01). Correlation analyses were performed using Pearson's method, reporting the correlation coefficient (r) as the effect size along with its 95% confidence interval. The correlation coefficients (r) ranged from − 1 to 1. r > 0 and < 0 represented positive and negative correlations, respectively. The |r| value denoted the degree of correlation among variables. In particular, r = − 1, 0, and 1 reflected a completely negative correlation, nocorrelation and a completely positive correlation, respectively.

## Results

### Effects of adding SY and VE on the growth, development and antioxidant immunity of lambs

**Fig. 1:**
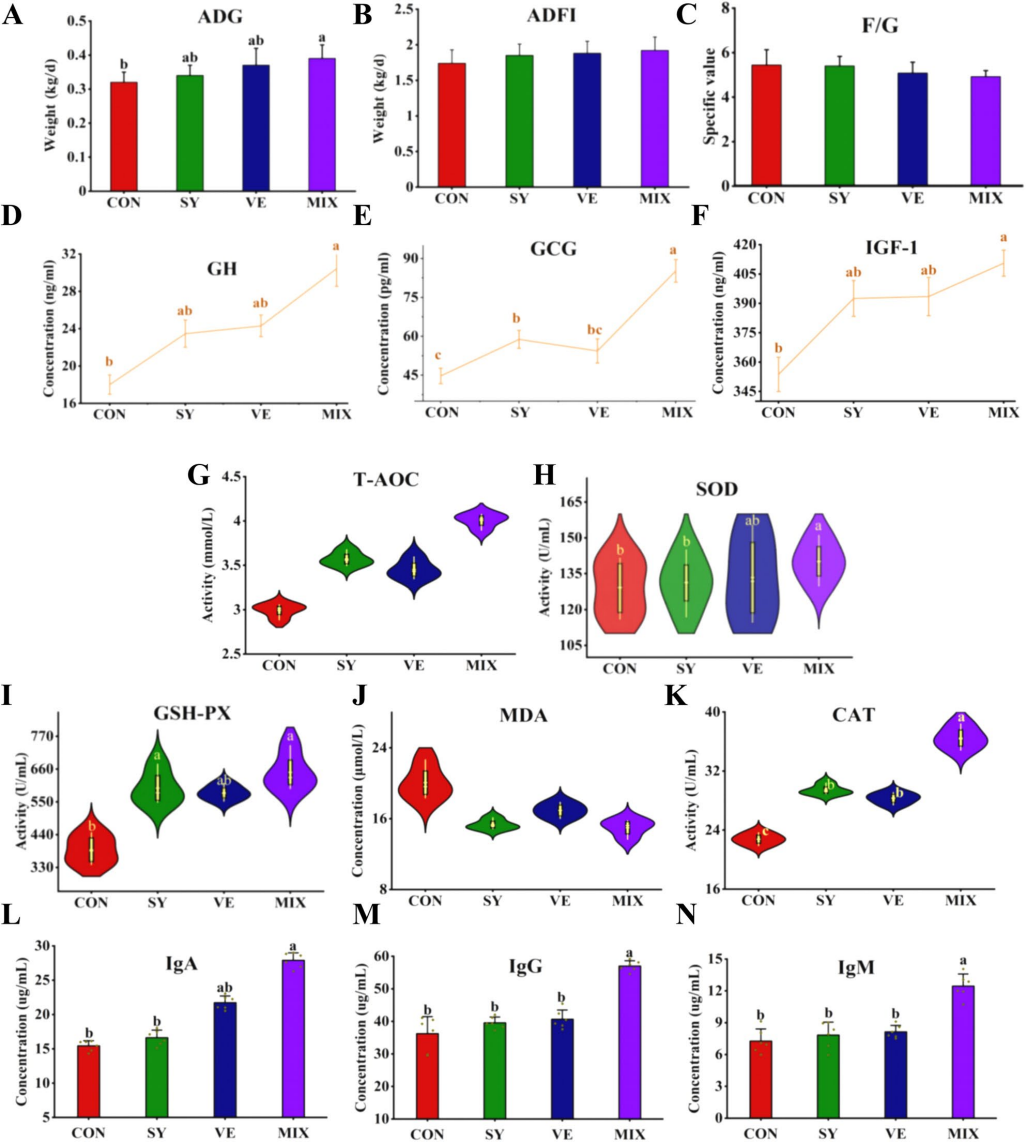
Analysis of growth and development, serum growth hormone ang antioxidant immune indicators of lambs. (**A**) ADG, (**B**) ADFI, (**C**) F/G, (**D**) GH, (**E**) GCG, (**F**) IGF-1, (**G**)T-AOC, (**H**) SOD, (**I**) GSH-PX, (**J**) MDA, (**K**) CAT, (**L**) IgA, (**M**) IgG, (**N**) IgM. The same lowercase letters (a, b) indicate no significant difference (*P* > 0.05), while different lowercase letters indicate a singnificant difference (*P* < 0.05)

Dietary supplementation with SY, VE, and their mixture (MIX) significantly affected the growth, antioxidant status, and immunity of lambs. Compared with the CON group, the MIX group showed the most pronounced improvements. Specifically, the ADG was significantly increased (*P* < 0.05), and the F/G exhibited the greatest reduction (*P* > 0.05) in the MIX group (Fig. 1). Concurrently, serum levels of GH and IGF-1 were significantly elevated in the MIX group (*P* < 0.05). Moreover, the GCG concentration in the MIX group was extremely significantly higher than that in the CON group (*P* < 0.01), and also significantly higher than that in both the SY and VE groups (*P* < 0.05).

Analysis of serum antioxidant parameters showed that SOD activity was significantly higher in the MIX group than in the CON and SY groups (*P* < 0.05). GSH-PX activity in the SY and MIX groups was significantly elevated compared with the CON group (*P* < 0.05). CAT activity in the MIX group was significantly higher than in the SY and VE groups, and was extremely significantly higher than in the CON group (*P* < 0.01) Regarding serum immune markers, the concentrations of IgA, IgG, and IgM in the MIX group were significantly higher than those in the CON, SY, and VE groups (*P* < 0.05), with the lowest levels observed in the CON group.

### Effects of adding SY and VE on rumen fermentation function and epithelial structure of lambs

Analysis of rumen fermentation parameters revealed that both rumen fluid pH (*P* = 0.014) and NH₃-N concentration (*P* = 0.013) were significantly reduced in the MIX group (Table 2). TVFA production was significantly higher in the SY and MIX groups than in the CON and VE groups (*P* < 0.05). Similarly, acetate and butyrate concentrations in these two groups (SY and MIX) were also significantly elevated compared with the CON and VE groups (*P* < 0.05).

**Table 2:** Rumen fermentation parameters of lambs

Item	Treatment				SEM	*P-Value*
	**CON**	**SY**	**VE**	**MIX**		
pH	6.68^a^	6.64^a^	6.68^a^	6.51^b^	0.022	0.014
NH_3_-N,mg/100 mL	10.20^a^	10.20^a^	9.10^ab^	8.32^b^	0.282	0.013
Total VFA,mmol/L	29.99^b^	36.53^a^	29.08^b^	38.56^a^	4.681	0.004
Acetic acid,mmol/L	17.10^b^	21.00^a^	18.23^b^	21.12^a^	1.972	0.014
Propionic acid,mmol/L	7.37	9.41	6.56	10.05	2.074	0.217
Isobutyric acid,mmol/L	0.72	0.65	0.56	0.80	0.127	0.250
Butyric acid,mmol/L	2.20^b^	3.38^a^	2.15^b^	3.49^a^	0.774	0.028
Isovaleric acid,mmol/L	1.36	1.33	1.01	1.49	0.217	0.121
Pentanoic acid,mmol/L	0.70	0.76	0.60	0.94	0.153	0.120
Acetic/Propionic ratio	2.33	2.23	2.79	2.10	0.130	0.320

The same lowercase letters ^a^, ^b^ indicate no significant difference* P* > 0.05, while different lowercase letters indicate a significant difference *P* < 0.05

**Fig. 2:**
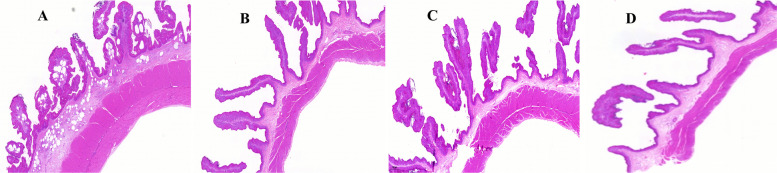
Histological features of rumen epithelium in lambs. (**A**) CON, (**B**) SY, (**C**) VE, (**D**) MIX. Cut at 500 μm

Histomorphometric analysis of the rumen epithelium (Fig. 2, Table 3) indicated that rumen papilla length was significantly greater in the MIX and VE groups than in the CON and SY groups (*P* < 0.05). In contrast, the thickness of the muscular layer was significantly lower in the SY and MIX groups compared with the CON and VE groups (*P* < 0.05). Furthermore, rumen papilla width in the MIX group was significantly increased relative to the CON group (*P* < 0.05).

**Table 3:** Histological morphological characteristics of rumen epithelium

Item	Treatment				SEM	*P-Value*
	**CON**	**SY**	**VE**	**MIX**		
Papilla length,µm	2289.14^b^	2318.41^b^	3257.56^a^	3167.46^a^	160.229	0.011
Papilla width,µm	449.85^b^	491.65^ab^	518.45^ab^	554.99^a^	50.127	0.075
Muscle thickness,µm	1125.35^a^	733.65^b^	1138.89^a^	857.41^b^	61.729	0.013

The same lowercase letters ^a^, ^b^ indicate no significant difference* P* > 0.05, while different lowercase letters indicate a significant difference* P* < 0.05

### Effects of adding SY and VE on the structure and function of rumen microorganisms in lambs

**Fig. 3:**
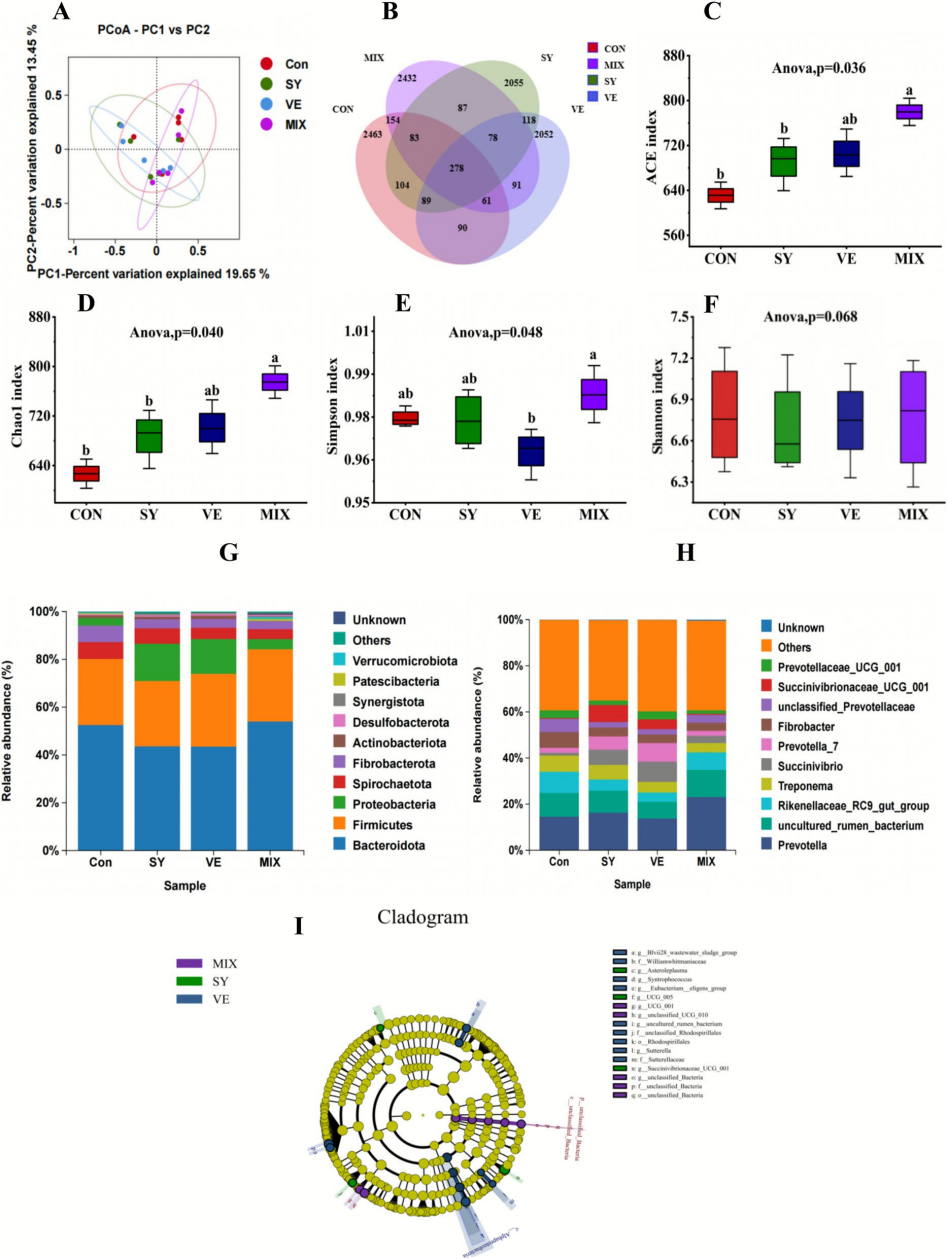
Analysis of the diversity and composition of rumen microorganisms in lambs. **A** PCoA analysis, **(B)** OTU-Venn analysis,**(C-F)** ACE, Chao, Simpson and Shannon indices, (**G**) phylum level microorganisms, (**H**) genus level microorganisms, (**I**) LEfSe analysis. The same lowercase letters (a,b) indicate no significant difference* P* > 0.05, while different lowercase letters indicate a significant difference* P* < 0.05

**Fig. 4:**
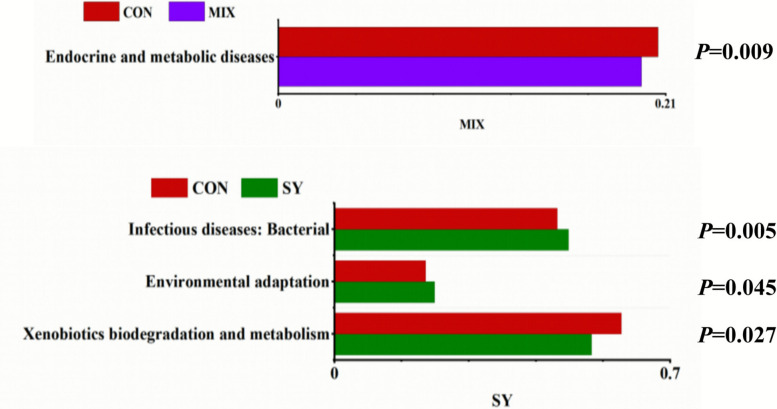
Functional analysis of differential microbiota KEGG in the rumen of lambs

Analysis of the rumen microbiota structure, as visualized by PCoA (Fig. 3), revealed distinct clustering patterns among the treatment groups. A total of 278 core operational taxonomic units (OTUs) were shared across all groups. The CON, SY, VE, and MIX groups contained 3322, 2892, 2857, and 3264 OTUs, respectively. Alpha diversity analysis indicated that the ACE and Chao1 indices were significantly higher in the MIX group than in the CON and VE groups. Moreover, the Simpson index of the MIX group was significantly higher than that of the VE group (P < 0.05). At the phylum level, *Bacteroidota* and *Firmicutes* were the two most dominant taxa across all groups. The relative abundance of *Firmicutes* in the MIX group was significantly higher than that in the CON group (*P* < 0.05). At the genus level, *Prevotella* and *uncultured_rumen_ bacterium* were predominant. The relative abundance of *Prevotella* was significantly elevated in the MIX group compared with the CON, SY, and VE groups (*P* < 0.05). Linear discriminant analysis Effect Size (LEfSe) identified several differential biomarkers (LDA score > 2.0) for each treatment group. The SY group was characterized by taxa such as *Asteroleplasma*, *UCG_005*, and *Succinivibrionaceae_UCG_001*; the VE group by *Blvii28_wastewater*, *Williamwhitmaniaceae*, and *Syntrophococcus*; and the MIX group by *UCG_001*, *unclassified_UCG*, and *unclassified_ Bacteria*. No significant biomarker was identified for the CON group. Functional prediction based on the KEGG database (Fig. 4) revealed that, compared with the CON group, pathways related to Endocrine and metabolic diseases were downregulated in the MIX group. In the SY group, pathways associated with Infectious diseases: Bacterial and Environmental adaptation were upregulated, whereas those involved in Xenobiotics biodegradation and metabolism were downregulated.

### Rumen microbial fermentation metabolism driven by SY and VE is related to lamb growth, antioxidation and immunity

**Fig. 5:**
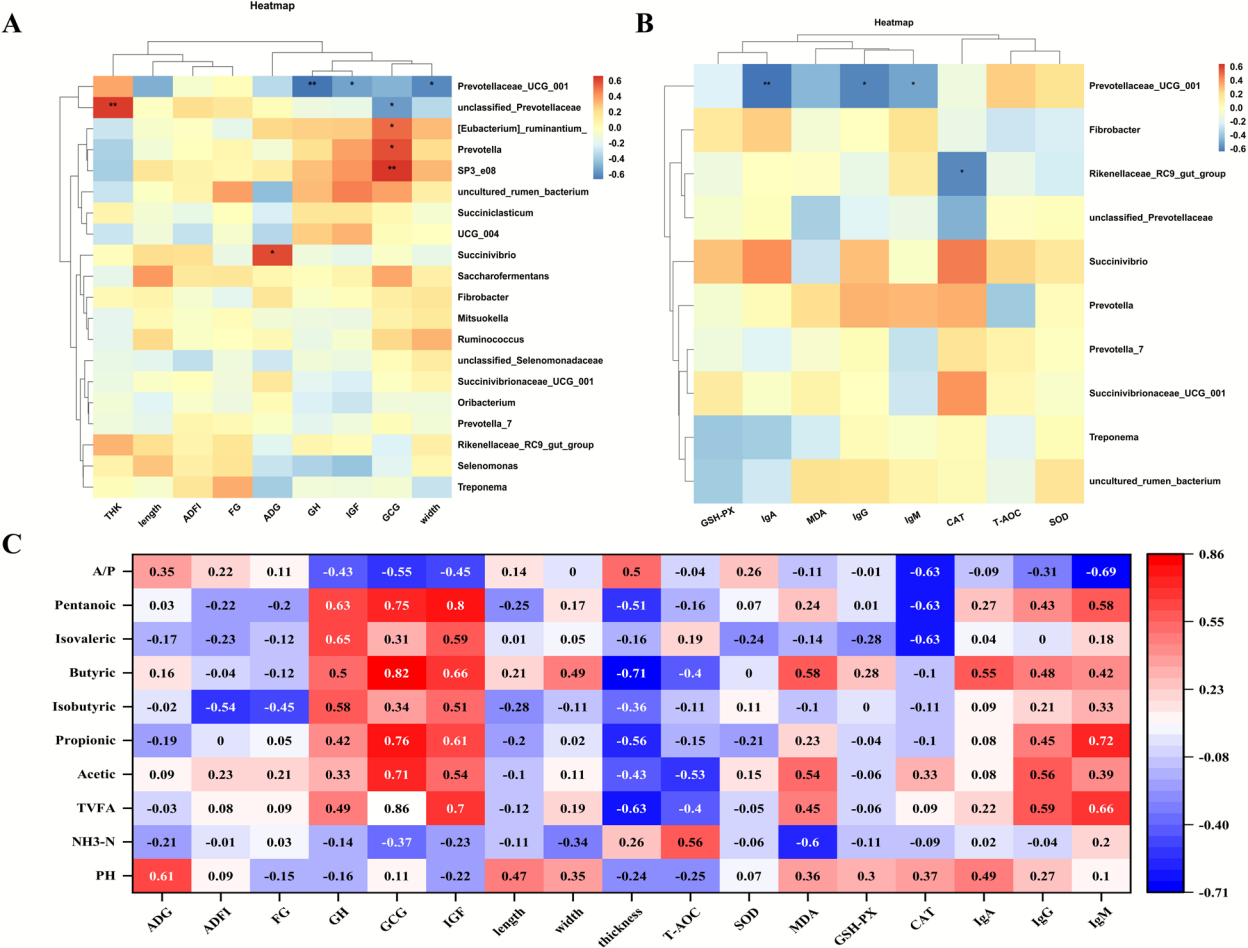
Correlation analysis of rumen microbial fermentation metabolism in lambs with their growth, antioxidation and immunity. *P < 0.05, **P < 0.01

To further elucidate the correlations between SY and VE induced changes in rumen microbial fermentation metabolism and lamb growth, antioxidant status, and immunity, correlation analysis was performed (Fig. 5 A and B). ADG showed a significant positive correlation with *Succinivibrio* (*P* < 0.05). The GCG was significantly positively correlated with the *[Eubacterium] ruminantium*_*group*, *Prevotella*, and *SP3-e08* (*P* < 0.05), whereas it exhibited a significant negative correlation with *Unclassified_Prevotellaceae* (*P* < 0.05). Both GH and IGF-1 were significantly negatively correlated with *Prevotellaceae_UCG-001* (*P* < 0.05).

Concerning immune parameters, the serum concentrations of IgA, IgG, and IgM were all significantly negatively correlated with *Prevotellaceae_UCG_001* (*P* < 0.05). Notably, IgM was significantly positively correlated with the *[Eubacterium]_ruminantium_group* (*P* < 0.05). For antioxidant indicators, CAT activity was significantly negatively correlated with *UCG_004*, *Rikenellaceae_RC9_gut_group*, and *Succiniclasticum* (*P* < 0.05), and significantly positively correlated with *Mitsuokella* (*P* < 0.05). Regarding rumen morphological traits, muscle thickness showed a significant positive correlation with *Unclassified_Prevotellaceae* (*P* < 0.05).

Rumen VFAs were significantly correlated with various phenotypic indices of lambs (Fig. 5C). Muscle thickness was significantly negatively correlated with the concentrations of TVFA, propionate, and butyrate (*P* < 0.05). ADG and IgA showed significant positive correlations with rumen pH (*P* < 0.05). T-AOC was positively correlated with NH₃-N concentration (*P* < 0.05), while MDA was negatively correlated with NH₃-N (*P* < 0.05). GCG, IGF-1, IgG, and IgM levels were significantly positively correlated with TVFA, acetate, propionate, isovalerate, and valerate (*P* < 0.05). GH and IGF-1 were positively correlated with isobutyrate (*P* < 0.05), in contrast to ADFI, which was negatively correlated with isobutyrate (*P* < 0.05). GH, GCG, IGF-1, and MDA were positively correlated with butyrate (*P* < 0.05). GH and IGF-1 were also positively correlated with isovalerate (*P* < 0.05). Conversely, GCG and IgM were significantly negatively correlated with the A/P (*P* < 0.05).

### Effects of adding SY and VE on the transcriptional levels of lamb livers

**Fig. 6:**
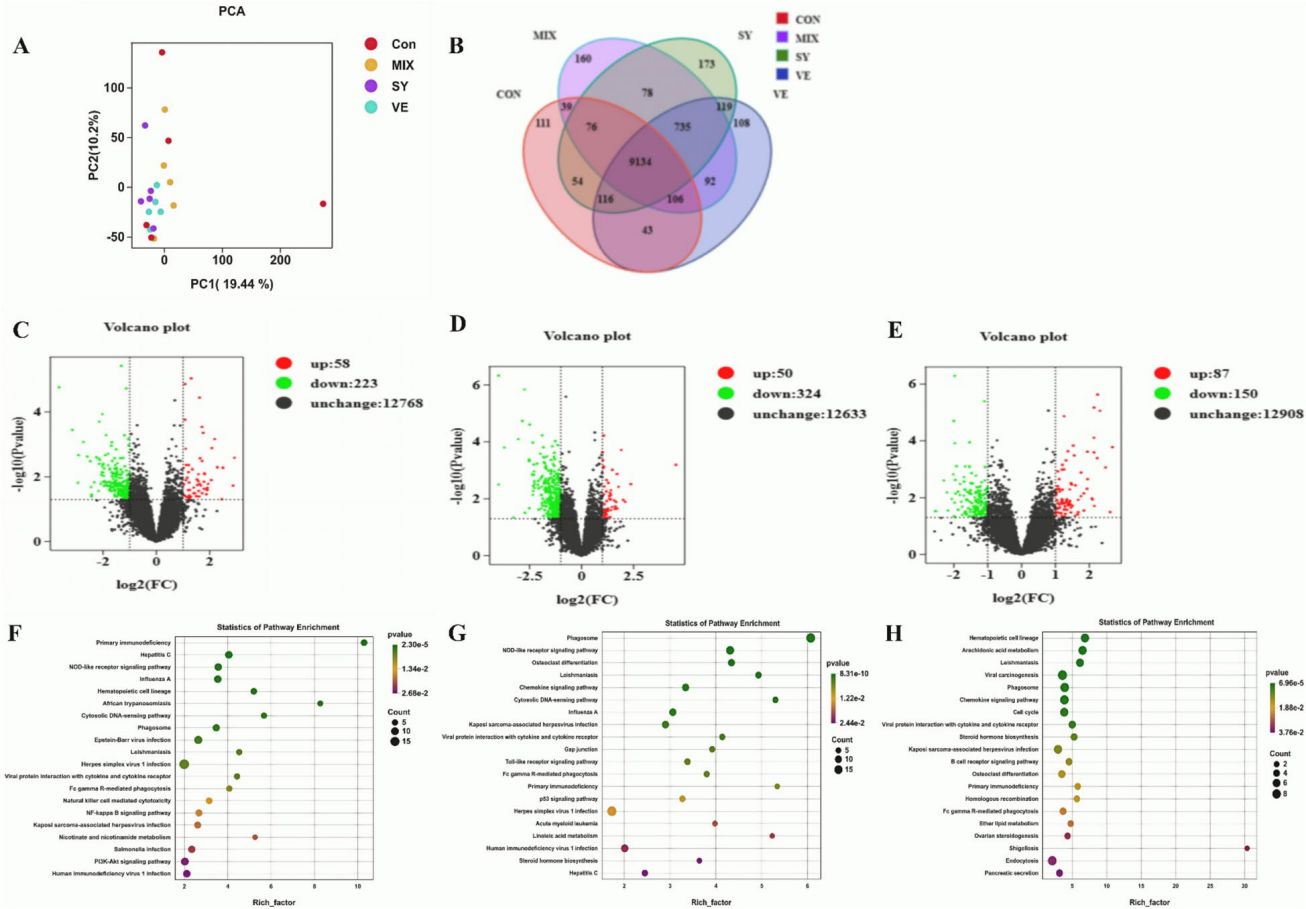
Transcriptome analysis of Lamb liver. **A** shows PCA, (**B**) is the Venn plot of gene expression, (**C-E**) are the volcano plots of differential gene expression between SY, VE and MIX and the CON group respectively, (**F–H**) are the KEGG enrichment plots of differential expression between SY, VE and MIX and the CON group respectively

Liver transcriptome analysis across the different treatment groups generated a total of 125.73 Gb of high-quality clean data. Each sample yielded an average of 5.81 Gb, with a Q30 base percentage exceeding 93.19%. Alignment of the sequences to the reference genome resulted in mapping efficiencies ranging from 91.63% to 96.58%. Differentially expressed genes (DEGs) were screened based on both effect size (|log FC|≥ 2) and statistical significance (*P* < 0.05) (Fig. 6). Principal component analysis (PCA) revealed distinct clustering patterns in gene expression profiles among the groups. A total of 9679, 10,485, 10,453, and 10,420 genes were identified in the CON, SY, VE, and MIX groups, respectively, with 9134 genes being commonly expressed across all four groups.Compared to the CON group, 58 genes were significantly upregulated and 223 were downregulated in the SY group. The VE group exhibited 50 upregulated and 324 downregulated genes. In the MIX group, 87 genes were upregulated and 150 were downregulated. Functional enrichment analysis of the differentially expressed genes revealed distinct pathway profiles among the groups. The SY group exhibited significant enrichment in pathways such as Primary immunodeficiency, African trypanosomiasis, and Nicotinate and nicotinamide metabolism. The VE group was enriched in Phagosome, Nitrogen metabolism, Primary immunodeficiency, and Linoleic acid metabolism. The MIX group showed primary enrichment in Arachidonic acid metabolism and Primary immunodeficiency, with Viral carcinogenesis being the most significantly enriched pathway across all comparisons.

### SY and VE regulate liver degenes and are associated with lamb growth, antioxidation and immunity

**Fig. 7:**
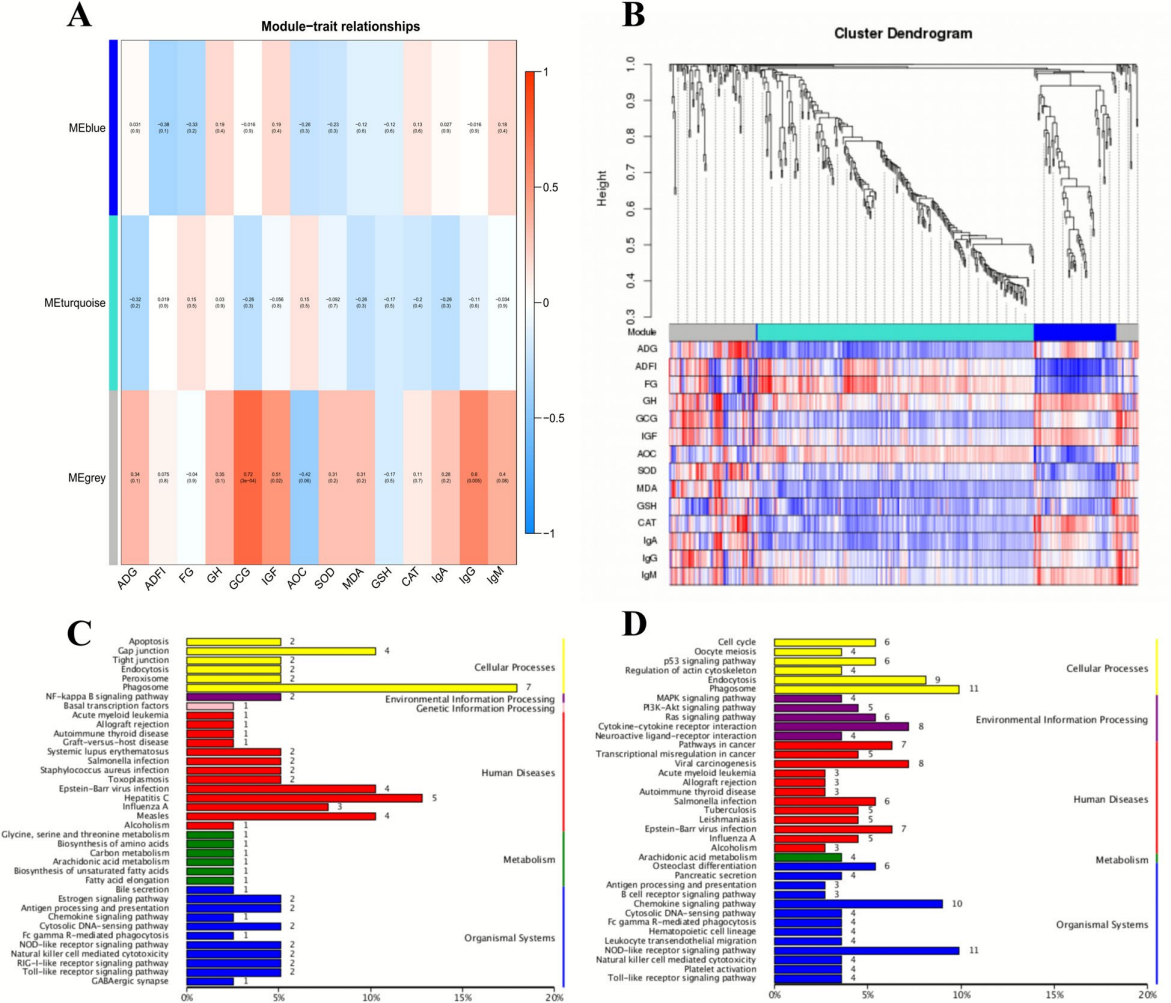
WGCNA analysis of differentially expressed genes and growth, antioxidant and immune indicators of lambs. **A** WGCNA module diagram, (**B**) Gene-phenotype clustering diagram, (**C**) KEGG enrichment diagram (Meblue), (**D**) KEGG enrichment (MEturquoise)

Weighted Gene Co-expression Network Analysis (WGCNA) was employed to identify clusters (modules) of highly co-expressed genes across all samples, independent of treatment groups. This systems biology method constructs a gene network based on pairwise correlation strengths, groups genes into modules using hierarchical clustering. This study is based on the liver transcriptome sequencing data, WGCNA was performed to identify modules of co-expressed genes and to explore their correlations with key phenotypic traits of lambs, including growth performance, antioxidant capacity, and immune function (Fig. 7). The analysis revealed that the MEgrey module exhibited a highly significant and strong positive correlation with serum IgG, GCG, and IGF-1 levels (*P* < 0.05). KEGG pathway enrichment analysis of DEGs within the identified modules further elucidated their functional relevance. DEGs in the MEblue module were significantly enriched in pathways involved in immune-metabolic crosstalk, notably Biosynthesis of amino acids, Carbon metabolism, Arachidonic acid metabolism, Biosynthesis of unsaturated fatty acids, and Bile secretion. Furthermore, DEGs in the MEturquoise module were predominantly enriched in processes related to secretory activity and immune regulation, including Pancreatic secretion, Arachidonic acid metabolism, Antigen processing and presentation, and the Toll-like receptor signaling pathway.

### SY and VE drive rumen microbiota-VFA-liver mRNA interactions in lambs

**Fig. 8:**
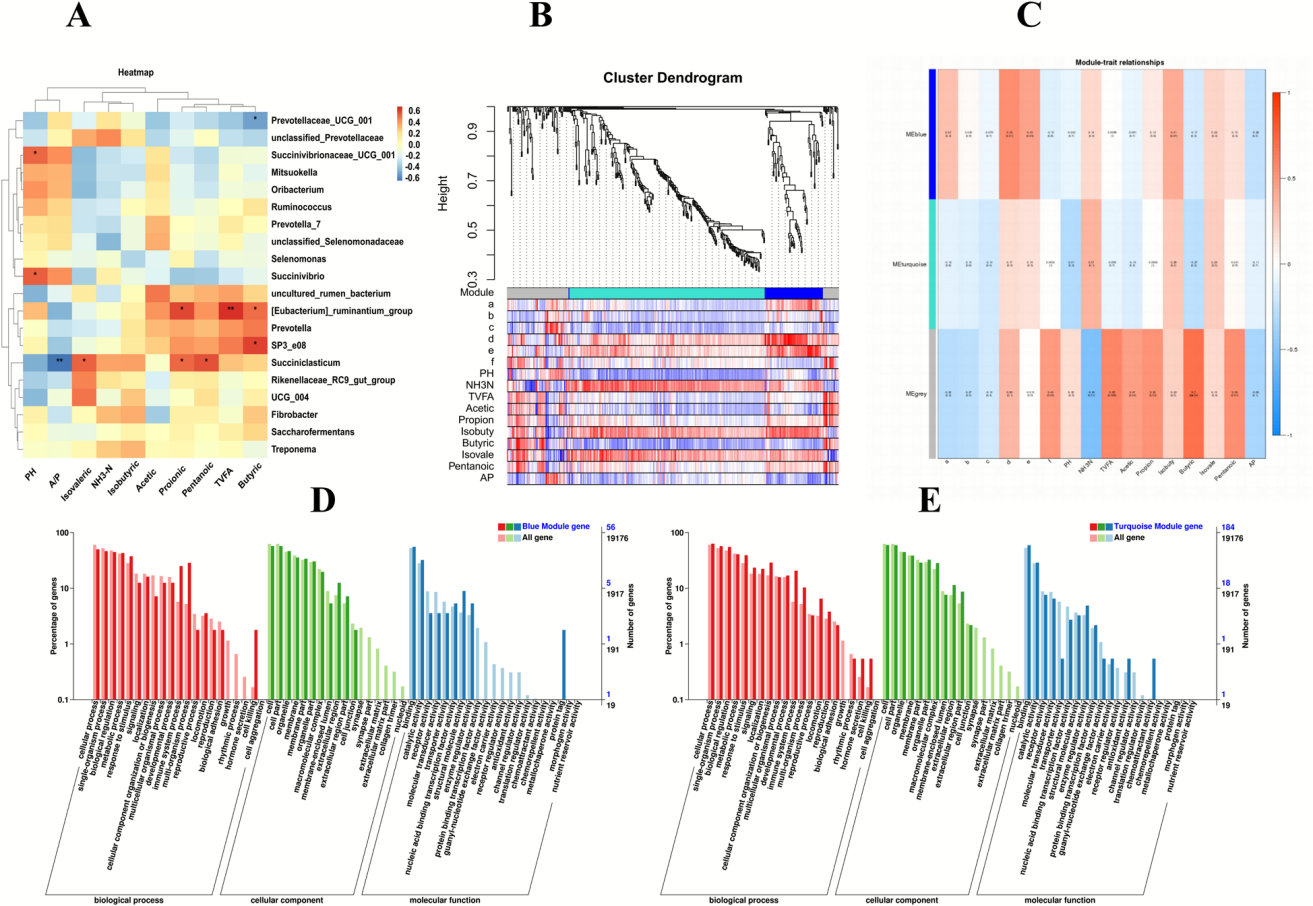
Analysis of the interaction between rumen microbiota, VFA and liver mRNA in lambs. **A** Heat map illustrating the correlation among the top 20 rumen bacterial genera and volatile fatty acid parameters. **B** Plot of WGCNA modules. **C** Gene-phenotype clustering map displaying (a: *Prevotellaceae_UCG_001*, b: *Succinivibrionaceae_UCG_001*, c: *Succinivibrio*, d: *[Eubacterium] _ruminantium_group*, e: *Succiniclasticum, and* f*: SP3_e08)*. **D** Plot of GO enrichment (Meblue). **E** Map of GO Enrichment (MEturquoise)

Through the interaction analysis of rumen microbiota—VFA-liver mRNA (Fig. 8), it was found that pH was significantly positively correlated with *Succinivibrionaceae_UCG_001* and *Succinivibrio* (*P* < 0.05). The A/P ratio showed a significant negative correlation with *Succiniclasticum* (*P* < 0.05). Concentrations of isovalerate, propionate, and valerate (pentanoate) were all significantly positively correlated with *Succiniclasticum* (*P* < 0.05). TVFA was significantly positively correlated with the* [Eubacterium]_ruminantium_group* (*P* < 0.05). Butyrate concentration was significantly positively correlated with the *[Eubacterium]_ ruminantium_group* and *SP3-e08* (*P* < 0.05), and significantly negatively correlated with *Prevotellaceae_UCG-001* (*P* < 0.05).

To further elucidate the associations among VFAs, their related functional microorganisms, and hepatic gene expression, WGCNA was performed by integrating these three data types. The analysis identified three core gene modules that were significantly associated with specific VFAs and corresponding microbial taxa. The MEgrey module was significantly correlated with *SP3_e08*, NH₃-N, TVFA, acetate, propionate, butyrate, and valerate (*P* < 0.05). The MEblue module showed a significant positive correlation with the *[Eubacterium]_ruminantium_group* and *Succiniclasticum* (*P* < 0.05). Subsequently, Gene Ontology (GO) functional enrichment analysis of the DEGs within the MEblue module (FDR < 0.05) revealed that these genes were primarily enriched in immune‑metabolism‑related functional terms, includi-ng immune system process, cell killing, and structural molecule activity.

## Discussion

This study systematically investigated the effects of dietary supplementation with SY, VE, and MIX on the growth performance, antioxidant and immune function, rumen fermentation and morphology, rumen microbiota, and liver transcriptome of lambs. The findings preliminarily reveal a potential interactive regulatory network involving the "rumen microbiota–VFA – liver mRNA" that underpins the observed enhancements in lamb growth and antioxidant immunity.

Improving growth performance, with ADG and F/G as key evaluation indexes of growth efficiency, is a core objective in livestock nutrition research [20]. In the present study, the most pronounced growth benefits were observed in the MIX group, which exhibited a significant increase in ADG and the greatest reduction in F/G. This improvement was accompanied by elevated serum levels of three key endocrine factors (GH, IGF-1, and GCG). GH and IGF-1 regulate animal growth by facilitating cell proliferation, differentiation, and protein synthesis [21], while GCG activates glucose metabolism enzymes and promotes hepatic glycogenolysis to provide energy substrates for growth [22], collectively providing endocrine support for enhanced performance. These phenotypic improvements are closely linked to synergistically enhanced systemic antioxidant capacity and immune function. The antioxidant system is a crucial barrier for maintaining cellular homeostasis, with SOD, GSH-PX, and CAT forming the major defense against reactive oxygen species [23, 24]. Consistent with this, the MIX group showed significantly higher activities of these enzymes, reflecting a synergistic antioxidant effect, which is aligned with previous findings that SY, as an essential component of GSH-PX, acts to directly enhance enzymatic antioxidant capacity [25]. VE as a lipophilic antioxidant, scavenges free radicals in lipid membranes to protect structural integrity [26, 27], thereby jointly maintaining redox homeostasis.Concomitantly, the MIX group exhibited the highest concentrations of immunoglobulins (IgA, IgG, IgM). This is likely attributed to the improved antioxidant environment, which mitigates oxidative damage to immune cells and promotes B lymphocyte proliferation and antibody secretion [28]. Collectively, the elevated growth-related hormones, enhanced antioxidant defenses, and improved immune function synergistically promoted lamb health and feed efficiency, ultimately translating to superior overall growth performance.

The phenotypic improvements are intrinsically linked to optimized rumen function. As the core organ for nutrient digestion in ruminants, the stability of rumen fermentation parameters and epithelial structure directly affects feed utilization [29]. The synergistic effect of SY and VE significantly optimized this process. In the MIX group, rumen fluid pH and NH3-N concentration significantly decreased, indicating an optimized fermentation environment. An appropriate rumen pH (e.g., 6.51 in the MIX group) maintains microbial activity and supports synergistic interactions between fibrolytic and acid-producing bacteria [30]. The reduced NH3-N suggests improved efficiency in microbial protein synthesis and reduced nitrogen waste [31], consistent with findings that selenium and VE can regulate nitrogen metabolism in sheep [32]. Crucially, the contents of TVFA, acetate, and butyrate were significantly increased. TVFA provides the main energy supply, acetate participates in fat synthesis, and butyrate serves as a substrate for ATP generation [33, 34], directly providing an energy basis for increased ADG. The enrichment of acetate and butyrate further promoted rumen epithelial development. Correspondingly, rumen papilla length and width increased significantly, expanding the absorption surface area, while a reduction in muscle layer thickness may decrease nutrient diffusion resistance [35]. This morphological optimization enhances the absorption efficiency of VFA and other nutrients. The combined effect of “improving the fermentation environment — increasing VFA yield — optimizing epithelial structure” explains the enhanced digestive and absorptive functions in the MIX group.

This functional optimization is fundamentally driven by a reshaped rumen microbial community and its functional synergy [36]. The synergistic effect of SY and VE significantly altered microbial community characteristics. The ACE and Chao1 indices in the MIX group were significantly higher than in the CON and VE groups, and the Simpson index was higher than in the VE group, indicating improved microbial richness, evenness, and community stability. OTU analysis revealed a larger number of unique OTUs in the MIX group, suggesting the introduction or enrichment of specific functional microorganisms. At the phylum level, the relative abundance of *Firmicutes* was significantly increased. *Bacteroidota* and *Firmicutes* are closely associated with carbohydrate degradation [37], and *Firmicutes* in particular contain numerous fiber-degrading and butyrate-producing bacteria, promoting cellulose breakdown and butyrate generation [38], which aligns with the increased TVFA and butyrate observed. At the genus level, *Prevotella* was significantly enriched. *Prevotella* species secrete various glycoside hydrolases to efficiently degrade starch and fiber [39], further verifying the enhanced feed digestion efficiency. LEfSe analysis revealed differential biomarkers (e.g., *UCG_001* and *unclassified_UCG_010*) in the MIX group that may be involved in VFA metabolism and antioxidant synthesis. The more singular microbial functions in the SY or VE alone groups underscore the superior, synergistic effect of their combination. KEGG analysis indicated that pathways related to "endocrine and metabolic diseases" were decreased in the MIX group, suggesting a reduced risk of metabolic disorders through microbial modulation, while the increase in "environmental adaptation" pathways in the SY group might relate to microbial stress adaptation [40].

To explain the mechanism of action of SY and VE at the molecular level, this study further carried out liver transcriptomic analysis. PCA analysis indicated that there was a significant segregation trend in the gene expression profiles among different treatment groups, which provided a molecular basis for explaining their different physiological effects. Analysis of differentially expressed genes (DEGs) revealed that the SY group and the VE group were mainly down-regulated genes, while the gap in the number of up-regulated and down-regulated genes in the MIX group narrowed. This indicates that the combination of the two may alleviate the transcriptional suppression of a single intervention, demonstrating functional complementarity and synergy, which is consistent with the conclusion of Sivertsen [41]. In the GO enrichment analysis, the VE group was specifically enriched in the "membrane encapsulation cavity" item, which was consistent with its role in scavenging membrane oxidation free radicals. The MIX group enriched the most genes in "biological regulation", further supporting the synergistic regulation of the two in biological processes. KEGG pathway enrichment revealed that the SY group was specifically enriched in "niacin and nicotinamide metabolism", which might be related to the promotion of NAD(P) + synthesis by SY and the enhancement of GSH-PX activity; [42]. The VE group is enriched in the "phagosome" pathway, enhancing macrophage-mediated innate immunity; The MIX group was significantly enriched in "arachidonic acid metabolism", a pathway that generates lipid mediators such as prostaglandins and leukotrienes, which play a core role in regulating inflammation and immunity [43], indicating that SY and VE may systematically coordinate antioxidant and immune homeostasis by regulating lipid signaling networks.

**Fig. 9:**
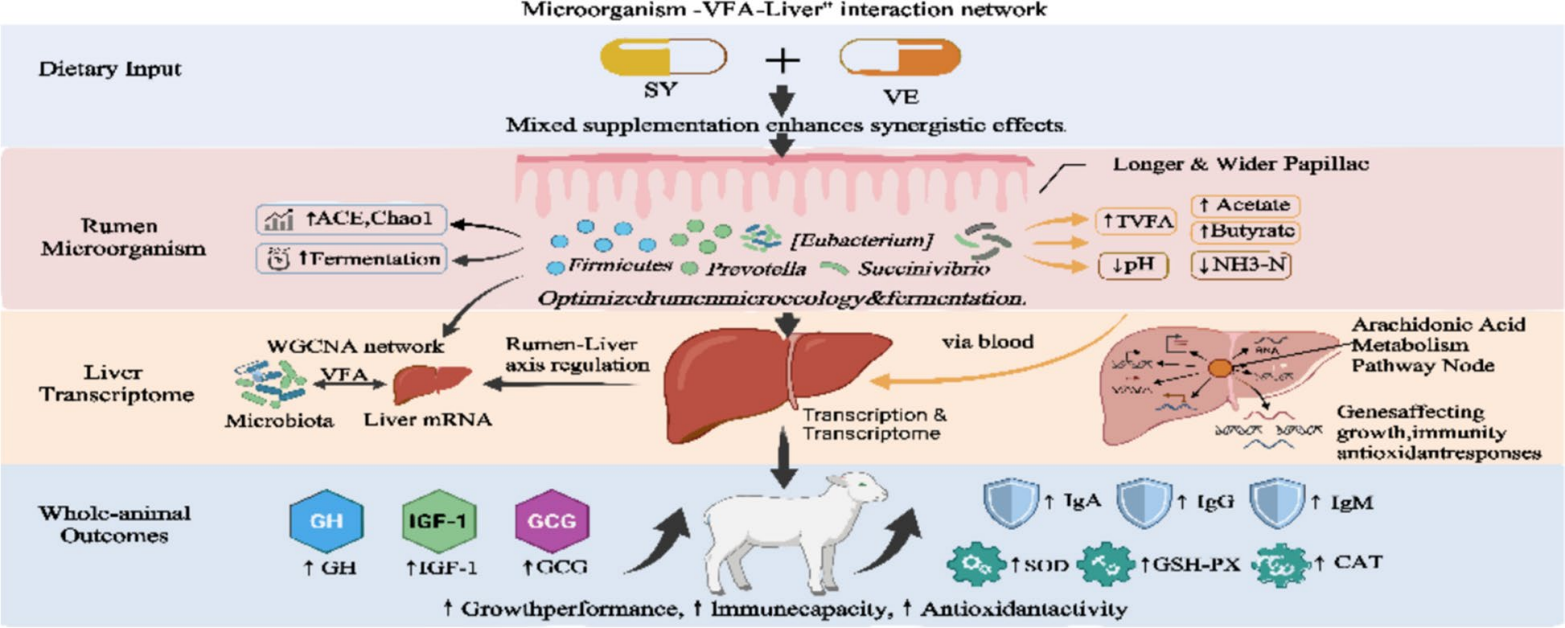
"Microorganism-VFA-Liver" regulatory mechanism diagram

The functional remodeling of rumen microorganisms further forms an interactive regulatory network with the host liver gene expression through the metabolic product VFA, jointly mediating the synergistic regulatory effect of SY and VE (Fig. 9). The results of correlation analysis indicated that rumen microbiota, VFA were associated with lamb growth, antioxidant and immune indicators. ADG was significantly positively correlated with Succinivibrio, and Succinivibrio could participate in the metabolism of succinic acid and propionic acid [44]. An increase in its abundance could promote the generation of VFA. And then provide energy for growth. GCG and IgM were significantly positively correlated with the [Eubacterium] ruminantium group. Studies have shown that this microbiota is related to immunity [45], and it may synergistically affect growth and immune function by regulating hormone secretion and antibody production. As a microbial metabolic product, VFA plays a key signaling role in the "microbiota-host" interaction. The significant positive correlation between TVFA, acetic acid, butyric acid, etc. in the MIX group and indicators such as GH, IGF-1, IgG indicates that VFA is not only an energy source, but also can regulate the endocrine system and the immune system. It affects the growth performance of lambs, which is related to Yu's discovery that supplementing selenium and vitamin E can improve growth performance and oxidative stress status [26]. As the metabolic and immune regulatory center, the liver's transcriptome response further explains the host's molecular adaptation to nutritional intervention. The differential genes in the MIX group were significantly enriched in pathways such as "hematopoietic cell lineage" and "arachidonic acid metabolism". WGCNA analysis further identified three gene modules closely related to growth and immune indicators. Among them, the MEgrey module had a strong correlation with IgG, GCG, IGF-1, etc., indicating that liver gene expression is directly involved in hormone and immune regulation.

This study has several limitations. First, the sample size was relatively limited, and future research with larger cohorts is needed to confirm these findings. Second, the experimental period was short; the long-term effects and economic viability of supplementation require further investigation. Third, the mechanistic insights are primarily correlative. Future studies employing in vitro models, metabolomic profiling, or targeted interventions are necessary to establish causality within the proposed network. Finally, the conclusions are based on a specific dietary background and breed; their generalizability to other feeding systems and genetic backgrounds warrants further evaluation.

## Conclusion

This study demonstrates that dietary supplementation with a combination of 0.6 mg SY and 200 IU VE constitutes an efficient nutritional regulation protocol for lambs, eliciting significantly superior effects compared to individual supplementation. The MIX treatment enhanced ADG and improved feed efficiency (F/G), which was associated with the up-regulated secretion of GH, IGF-1, and GCG. Concurrently, it synergistically enhanced systemic antioxidant capacity and immune function, suggesting the establishment of a positive “growth–antioxidant–immunity” cycle. Regarding rumen function, MIX optimized the fermentation environment and improved nutrient absorption efficiency, as evidenced by promoted rumen papilla development and reduced muscular layer thickness. The underlying mechanism appears to involve the synergistic remodeling of the rumen microbiota by SY and VE (e.g., enriching functional taxa such as *Firmicutes* and *Prevotella*), and our integrated analysis suggests the important involvement of an interactive “Microorganism–VFA–Liver” network in the coordinated regulation of growth, metabolism, and immunity observed in response to the combined supplementation.

## Data Availability

All the raw data were submitted to the NCBI Sequence Read Archive (SRA) database (Accession number: PRJNA1404346).

## Abbreviations

SY: Selenium yeast
VE: Vitamin E
ADG: Average daily gain
ADFI: Average daily feed intake
GH: Growth hormone
IGF-1: Insulin growth factor-1
GCG: Glucagon
T-AOC: Total antioxidant capacity of serum
GSH-PX: Glutathione peroxidase
SOD: Superoxide dismutase
MDA: Malondialdehyde
CAT: Catalase
F/G: Material-to-weight ratio
NH3-N: Ammonia nitrogen
TVFA: Total volatile fatty acid
VFA: Volatile fatty acid
OTUs: Operational taxonomic units
LEfSe: LDA effect size
PcoA: Principal coordinates analysis
KEGG: Kyoto Encyclopedia of Genes and Genomes
